# Proton beam therapy for gliomas: a multicenter prospective registry study from all proton beam facilities in Japan

**DOI:** 10.1093/jrr/rrac103

**Published:** 2023-04-07

**Authors:** Takashi Mori, Masashi Mizumoto, Katsuya Maebayashi, Kentaro Nishioka, Yoshiki Arakawa, Kazuhiko Kurozumi, Koichi Yasuda, Taisuke Sumiya, Hiroyasu Tamamura, Yoshitaka Sato, Takahiro Waki, Masaru Takagi, Yu Takada, Tomoaki Okimoto, Masao Murakami, Yasuhiro Kikuchi, Kazufumi Okada, Yoichi M Ito, Tetsuo Akimoto, Hidefumi Aoyama

**Affiliations:** Department of Radiation Oncology, Hokkaido University Hospital, Sapporo, Hokkaido 060-8638, Japan; Department of Radiation Oncology, University of Tsukuba, Tsukuba, Ibaraki 305-8575, Japan; Division of Radiation Oncology, Nippon Medical School Hospital, Tokyo 113-8602, Japan; Global center for Biomedical Science and Engineering, Faculty of Medicine, Hokkaido University, Sapporo, Hokkaido 060-8638, Japan; Department of Neurosurgery, Kyoto University Graduate School of Medicine, Kyoto 606-8507, Japan; Department of Neurosurgery, Hamamatsu University School of Medicine, Hamamatsu, Shizuoka 431-3192, Japan; Department of Radiation Oncology, Hokkaido University Hospital, Sapporo, Hokkaido 060-8638, Japan; Department of Radiation Oncology, University of Tsukuba, Tsukuba, Ibaraki 305-8575, Japan; Proton Therapy Center, Fukui Prefectural Hospital, Fukui, Fukui 910-8526, Japan; Proton Therapy Center, Fukui Prefectural Hospital, Fukui, Fukui 910-8526, Japan; Department of Radiology, Tsuyama Chuo Hospital, Tsuyama, Okayama 708-0841, Japan; Proton Therapy Center, Sapporo Teishinkai Hospital, Sapporo, Hokkaido 065-0033, Japan; Proton Therapy Center, Sapporo Teishinkai Hospital, Sapporo, Hokkaido 065-0033, Japan; Department of Radiology, Hyogo Ion Beam Medical Center, Tatsuno, Hyogo 679-5165, Japan; Department of Radiation Oncology, Southern Tohoku Proton Therapy Center, Koriyama, Fukushima 963-8563, Japan; Department of Radiation Oncology, Southern Tohoku Proton Therapy Center, Koriyama, Fukushima 963-8563, Japan; Data Science Center, Promotion Unit, Institute of Health Science Innovation for Medical Care, Hokkaido University Hospital, Sapporo, Hokkaido 060-8638, Japan; Data Science Center, Promotion Unit, Institute of Health Science Innovation for Medical Care, Hokkaido University Hospital, Sapporo, Hokkaido 060-8638, Japan; Department of Radiation Oncology, National Cancer Center Hospital East, Kashiwa, Chiba 277-8577, Japan; Department of Radiation Oncology, Faculty of Medicine and Graduate School of Medicine, Hokkaido University, Sapporo, Hokkaido 060-8638, Japan

**Keywords:** proton beam therapy (PBT), glioblastoma, glioma, proton-net

## Abstract

We reviewed the outcomes of glioma patients enrolled in a prospective observational registry study of proton beam therapy (PBT) in Japan. The inclusion criteria were glioma patients registered in the Electronic Data Capture system, the Proton-net, between May 2016 and July 2019. Data on patient characteristics, treatments, late adverse events, survival status, recurrence and secondary tumors were extracted and statistically analyzed. The primary endpoint was the overall survival (OS) rate, and the secondary endpoints were the progression-free survival (PFS) rate and cumulative local recurrence rate (cLRR). Of the 65 primary brain tumor patients registered, 29 glioma patients from eight of 19 PBT facilities met the inclusion criteria. There were 19 glioblastoma patients, eight of other malignant gliomas, and two of low-grade gliomas. For glioblastomas, with a median follow-up period of 16 months, the median survival time was 21.2 months and the OS at 1, 2, 3 and 4 years were 77.4%, 44.9%, 23.9% and 23.9%, respectively. The median PFS period was 10.1 months, the 1, 2, 3 and 4-year PFS were 32.4%, 19.4%, 9.7% and 9.7%, respectively. The 1, 2, 3 and 4-year cLRR were 56.1%, 68.8%, 78.4 and 78.4%, respectively. Grade 3 brain necrosis was observed in two patients. No secondary tumor was observed. This is the first report on the current status of PBT for gliomas in Japan. For glioblastomas, the outcomes of PBT are estimated to be equivalent to historical data of photon therapy. The results of a prospective comparative evaluation of PBT and photon therapy are awaited.

## INTRODUCTION

The glioma is a primary brain tumor thought to arise from glial cells, the supporting tissue of neurons, and gliomas account for about 27% of primary brain tumors [1]. In the WHO classification, gliomas are distinguished into Grades I to IV from a combination of histopathologic grade and prognosis data. Grade I and II gliomas are referred to as low-grade gliomas, and Grade III and IV gliomas are collectively referred to as malignant gliomas and have often been treated under a single designation in previous clinical studies. Surgery is the main approach to treatment for malignant gliomas, however, residuals of tumors are common due to the highly invasive nature of them. Postoperative radiotherapy is now the standard treatment for malignant gliomas because several clinical trials around 1980 showed that postoperative radiotherapy significantly improves the prognosis compared with supportive care alone or adjuvant chemotherapy only [2–5]. In a phase III trial comparing concurrent radiotherapy and temozolomide followed by maintenance chemotherapy and radiotherapy alone with a dose of 60 Gy in 30 fractions, a significant improvement in prognosis was observed in the radiotherapy and temozolomide group, which has now become used as the standard treatment for glioblastomas in adults [6, 7]. In addition, tumor-treating fields systems to maintenance chemotherapy improves the prognosis of newly diagnosed glioblastoma patients [8]. Despite these multimodality treatments, the prognosis of glioblastomas is still poor.

In the area of radiotherapy, there have been attempts to increase doses by using the intensity-modulated radiation therapy (IMRT) technique or adding stereotactic radiotherapy, and also alternative dose fractionation, but these have not resulted in improved outcomes. Proton beam therapy (PBT) has a Bragg peak and can reduce the dose to the normal tissue surrounding the tumor. At present four randomized controlled trials comparing PBT with X-ray therapy for malignant gliomas are currently ongoing, but only a few retrospective studies in single institutions have been reported.

In Japan, PBT for primary brain tumors, including malignant and low-grade gliomas, is provided within the framework of advanced medical care, and brain tumors are included in the targeted diseases of the multicenter, single-arm prospective observational study on PBT conducted since 2016. In this present study, we analyzed the treatment outcomes of malignant gliomas, especially glioblastomas, at multiple centers in Japan and evaluated the efficacy and safety of PBT for malignant gliomas by comparing the results with the standard results of treatment with photon therapy.

## MATERIALS AND METHODS

### Patients and treatment

This study was performed by the Central Nervous System Tumor Working Group in the Particle Beam Therapy Committee and Subcommittee at the Japanese Society for Radiation Oncology (JASTRO) and approved by the Hokkaido University Hospital Clinical Research and Medical Innovation Center in August 2016 and registered at the UMIN Clinical Trials Registry as UMIN000022917. The inclusion criteria were as follows: brain glioma patients registered in the Electronic Data Capture system, the so-called Proton-net, between May 2016 and July 2019, first-time PBT cases, without dissemination or multiple cancers other than low-grade gliomas. In principle, radiation doses were in accordance with the JASTRO treatment policy of proton therapy and in this study the relative biological effectiveness (RBE) of proton beams is assumed to be uniformly 1.1: 60 Gy (RBE) in 30 fractions or a total dose of 96.6 Gy (RBE) in 56 fractions (twice daily, 50.4 Gy in 28 fractions of photon therapy and 23.1 Gy [RBE] in 14 fractions of PBT) was given for glioblastoma. High-grade gliomas other than glioblastoma and low-grade gliomas were treated with a total dose of 60 Gy (RBE) and 54Gy (RBE) in 30 fractions, respectively. Information on patient characteristics, diseases, PBT, concomitant chemotherapy, late adverse events of grade 3 or greater in the Common Terminology Criteria for Adverse Events (CTCAE) version 4.03, survival status, recurrence and secondary tumors registered in the Proton-net were extracted and statistically analyzed.

### Statistical analysis

The primary endpoint was the overall survival (OS) rate, and the secondary endpoints were the annual progression-free survival (PFS) rate and cumulative local recurrence rate (cLRR). Local recurrence is defined as either tumor recurrence from within the irradiated field or regrowth of the residual tumor. Tumor progression is defined as the occurrence of one or more of the following conditions: local recurrence, field recurrence, or distant metastasis. The event time variable was defined as the period from the date of the start of PBT to the date of the event or censoring. The safety endpoints were defined as adverse events of CTCAE grade 3 or higher and second cancer development. Two-tailed statistical hypothesis tests were performed at a significance level of 0.05, and two-tailed confidence intervals with a confidence coefficient of 95% were estimated. Based on the Kaplan–Meier method, we estimated the survival function and each point confidence interval based on the complementary log–log transformation at 1, 2 and 3 years. For the annual local control rate, we estimated the cumulative incidence function and point confidence intervals based on the complementary log–log transformation at 1, 2 and 3 years, with death before local recurrence as the competing risk. The SAS version 9.4 (SAS Institute Inc., Cary, NC, USA) was used in the analysis.

## RESULTS

Of the 65 primary brain tumor patients registered with the Proton-net between May 2016 and July 2019, 29 glioma patients from eight of 19 PBT facilities met the inclusion criteria. All cases were reviewed by the tumor boards, in which multiple experts participate, to determine whether PBT was indicated. The characteristics of all glioma patients are shown in Table 1: male 13/female 16, median age 56 years (range 31–80 years), ECOG PS 0/1/2/3/unknown were 14/8/5/1/1. Neurological symptoms were present in 13 and absent in the other 16. There were 19 patients of glioblastomas, eight patients of malignant gliomas other than glioblastomas, and two patients of low-grade gliomas. The discrepancy between the histopathologic and clinical diagnosis is detailed. Histological diagnosis was made in 27 of 29 patients. One of the 19 patients of glioblastoma had a tumor in the corpus callosum and was clinically diagnosed as glioblastoma without surgery, including biopsy. Similarly, one patient of brainstem glioma was clinically diagnosed as diffuse intrinsic pontine glioma without biopsy. Two patients with pathologically diagnosed oligodendrogloima and diffuse astrocytoma were diagnosed clinically as malignant gliomas based on the rate of growth and the presence of enhancing effects on magnetic resonance imaging, although biopsy was the only procedure available and the pathological diagnosis was Grade II glioma. The only case of oligodendroglioma and one case each of anaplastic oligodendroglioma and anaplastic astrocytoma were diagnosed according to the 3rd edition of the WHO Classification of Tumors of the Central Nervous System, while the other cases were diagnosed according to the 4th edition. Surgical results were: total resection in seven, partial resection in 13, biopsy in six and no surgery was performed with three patients. The O^6^-methylguanine-DNA methyltransferase (MGMT) promoter methylation was present in one, absent in two and unknown in 26 patients; there were IDH mutations in six, wild type in 10 and unknown in 13. Twenty-two patients received a total dose of 60 Gy (RBE) in 30 fractions, and three patients received a total dose of 96.6 Gy (RBE) in 56 fractions. One patient of clinically malignant glioma with a pathological diagnosis of oligodendroglioma received a total dose of 59.4 Gy (RBE) in 33 fractions. A patient with brain stem glioma received a total dose of 54 Gy (RBE) in 30 fractions. Of the two patients of diffuse astrocytomas, one received a total dose of 54 Gy (RBE) in 30 fractions and the other 50.4 Gy (RBE) in 28 fractions because of the tumor’s proximity to the optic chiasm. Temozolomide was used concomitantly in 22 patients, bevacizumab in one patient with recurrent glioblastoma and the combination of procarbazine, nimustine and vincristine (PAV) in an anaplastic oligodendroglioma patient. In the two patients of clinically malignant glioma with a pathological diagnosis of diffuse astrocytoma and oligodendroglioma, the diffuse astrocytoma patient was treated with concurrent temozolomide, and the oligodendroglioma patient was treated with PAV therapy after PBT.

**Table 1:** Characteristics of the all glioma patients

Characteristics		*n* = 29
Gender	Male	13
	Female	16
Age, median (range)		56 (31–80)
ECOG PS	0	14
	1	8
	2	5
	3	1
	Unknown	1
Neurological symptom	present	13
	absent	16
Pathological diagnosis	Glioblastoma	18
	Anaplastic oligodendroglioma	3
	Anaplastic astrocytoma	2
	Diffuse astrocytoma	3
	Oligodendroglioma	1
	Not performed	2
Surgery	Gross total resection	7
	Partial resection	13
	Biopsy	6
	No surgery	3
Chemotherapy	Temozolomide	22
	Procarbazine, Nimustine, Vincristine	1
	Bevacizumab	1
	No chemotherapy	5
Dose	60 Gy (RBE)/30 Fr	22
	96.6 Gy (RBE)/56 Fr	3
	54 Gy (RBE)/30 Fr	2
	59.4 Gy (RBE)/33 Fr	1
	50.4 Gy (RBE)/28 Fr	1

ECOG PS: the Eastern Cooperative Oncology Group Performance Status

**Fig. 1:**
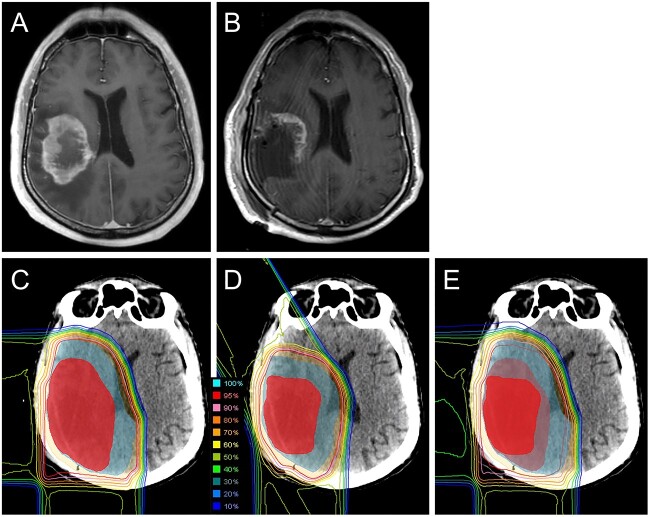
Magnetic resonance images and dose distribution for a patient with glioblastoma of the right frontal lobe. Postcontrast T1-weighted magnetic resonance imaging (MRI) before (A) and after surgery (B). Dose distributions of initial phase with 50 Gy (RBE) in 25 fractions (C), boost with 10 Gy (RBE) in five fractions (D) and the sum of the two with 60 Gy (RBE) in 30 fractions (E).

The representative case is shown in Fig. 1. This patient was a 56-year-old male with a partially resected glioblastoma of the frontal lobe who was treated with 50 Gy (RBE) in 25 fractions and 10 Gy (RBE) in five fractions of boost.

**Fig. 2:**
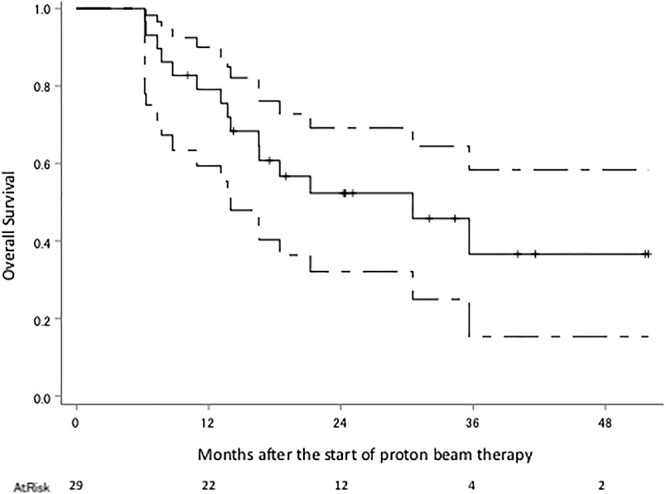
OS of the all glioma patients. The solid line shows the estimated value, and the upper and lower single-dotted lines show the upper and lower limits of the 95% confidence interval, respectively.

**Fig. 3:**
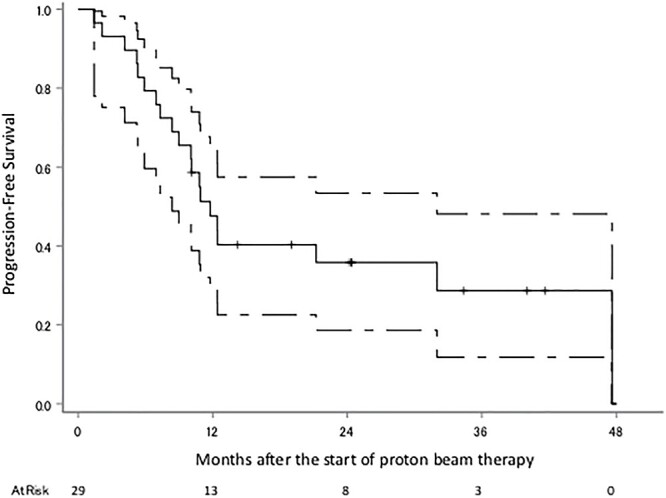
PFS of the all glioma patients. The solid line shows the estimated value, and the upper and lower single-dotted lines show the upper and lower limits of the 95% confidence interval, respectively.

**Fig. 4:**
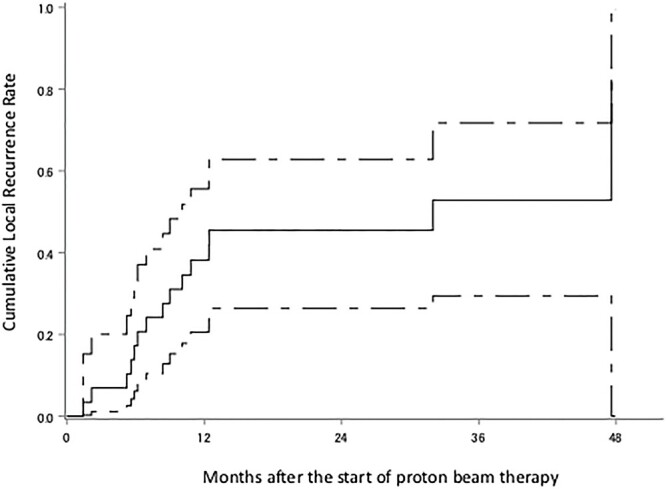
cLRR of the all glioma. The solid line shows the estimated value, and the upper and lower single-dotted lines show the upper and lower limits of the 95% confidence interval, respectively.

The median follow-up period was 18 months (range 6–51). Eighteen patients were diagnosed with recurrence, 11 of these with recurrence within the irradiated field, two without the irradiated field, three with local tumor regrowth, one within and without the irradiated field and one with intracranial dissemination; there were nine patients with no recurrence, and the status of the two remaining patients is unknown. The survival status at the last follow-up were: seven alive without tumors, five alive with tumors, 12 dead due to gliomas, two dead due to unknown causes and three were censored. Kaplan–Meier plots of OS, PFS and cLRR of all glioma patients are shown in Figs 2–4 respectively. The median survival time was 30.5 months (lower limit of 95% confidence interval 14.0 months), and the 1, 2, 3 and 4-year OS rates were 79.2% (95% confidence interval 59.4–90.1), 52.4% (32.1–69.2), 36.6% (15.3–58.4) and 36.6% (15.3–58.4), respectively. The median PFS period was 11.8 months (8.4–32.0), and 1, 2, 3 and 4-year PFS rate were 47.6% (28.7–64.3), 35.8% (18.6–53.4), 28.7% (11.8–48.1) and 0%, respectively. The 1, 2, 3 and 4-year cLRR were 38.2% (20.6–55.6), 45.5% (26.3–62.9), 52.9 (29.5–71.7) and 82.3% (0–99.5), respectively.

**Fig. 5:**
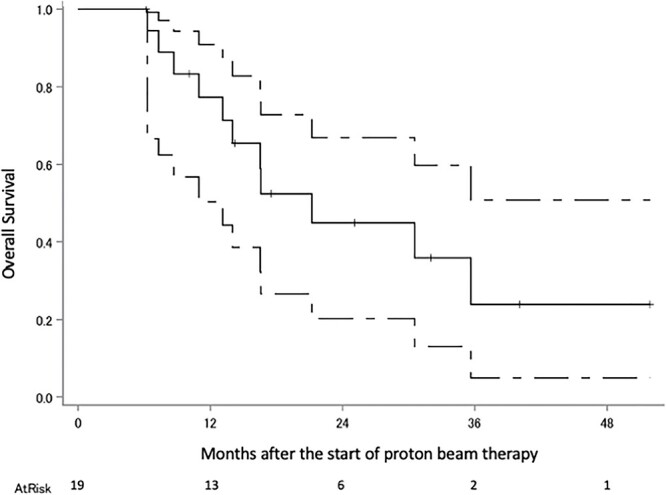
OS of glioblastoma patients. The solid line shows the estimated value, and the upper and lower single-dotted lines show the upper and lower limits of the 95% confidence interval, respectively.

**Fig. 6:**
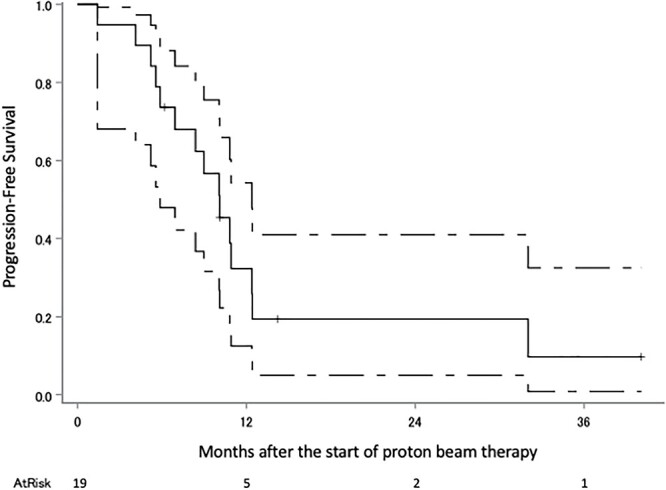
PFS of glioblastoma patients. The solid line shows the estimated value, and the upper and lower single-dotted lines show the upper and lower limits of the 95% confidence interval, respectively.

**Fig. 7:**
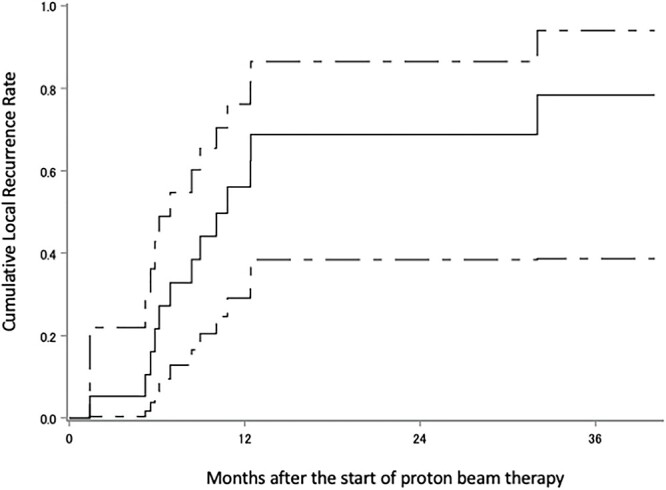
cLRR of glioblastoma patients. The solid line shows the estimated value, and the upper and lower single-dotted lines show the upper and lower limits of the 95% confidence interval, respectively.

Only the 19 patients of glioblastomas were statistically analyzed because the number of patients with other gliomas is too small for statistical analysis. Kaplan–Meier plots of OS, PFS and cLRR of glioblastoma patients are shown in Figs 5–7 respectively. The median survival time was 21.2 months (lower limit of 95% confidence interval 13.1 months), and the 1, 2, 3 and 4-year OS rates were 77.4% (95% confidence interval 50.3–90.9), 44.9% (20.3–66.9), 23.9% (4.9–50.9) and 23.9% (4.9–50.9), respectively. The median PFS period was 10.1 months (5.9–12.4), and 1, 2, 3 and 4-year PFS rate were 32.4% (12.5–54.3), 19.4% (5.0–40.9), 9.7% (0.8–32.6) and 9.7% (0.8–32.6), respectively. The 1, 2, 3 and 4-year cLRR were 56.1% (29.3–76.2), 68.8% (38.4–86.4), 78.4 (38.7–94) and 78.4% (38.7–94), respectively.

A Grade 3 or higher late adverse event was observed in two patients, both cases of central nervous system brain necrosis. One case was treated with hyperfractionated radiotherapy with a combination of X-rays and protons, 96.6 Gy (RBE) in 56 fractions. The other case was treated with 20 Gy in 10 fractions of X-ray therapy and 40 Gy (RBE) in 20 fractions of PBT. There was no evidence of secondary malignancies here.

## DISCUSSION

This is an observational study, and also reports the results of PBT for gliomas at multiple centers in Japan for the first time. We identified 29 glioma cases, of which glioblastomas accounted for the majority (19 cases). This is similar to the cohort in photon therapy [9]. Although the number of gliomas other than glioblastomas was very small and statistical evaluation could not be performed, these details are significant to fully show the status quo of PBT for malignant gliomas in Japan.

The Brain Tumor Registry of Japan (2005–2008) reported that over a four-year period, 1774 of 2049 (87%) glioblastomas and 819 of 961 (85%) Grade III gliomas in the 2007 edition of the WHO Classification have received radiation therapy [1]. Considering that there are about 440 and 200 glioblastoma and Grade III glioma patients who receive radiotherapy per year, the data from this three-year national registry of all PBT cases in Japan indicate that only a small proportion of patients with malignant gliomas in Japan receive PBT. The results of the EORTC-NCIC phase III trial are well known as the benchmark for the outcomes of radiotherapy with X-rays for glioblastomas. In this study, 60 Gy in 30 fractions radiotherapy were administered using 3-dimensional conformal radiation therapy (3D CRT) with X-rays of 6 MV or higher energies in combination with temozolomide. The median survival time for these cases was 14.6 months, and the OS at 2, 3, 4 and 5 years were 27.2%, 16.0%, 12.1% and 9.8%, respectively. The 2-, 3-, 4- and 5-year PFS were 11.2%, 6.0%, 5.6% and 4.1%, respectively [6, 7]. The Japan Clinical Oncology Group clinical trial for glioblastomas (JCOG0911) also used 3D CRT with 60 Gy in 30 fractions in combination with temozolomide, as in the EORTC-NCIC Phase III trial. In this trial, the median survival time was 20.3 months, the 1-, 2- and 3-year OS were 76.2%, 42.9% and 30.8%, respectively and the 1-, 2- and 3-year PFS were 38.1%, 26.9% and 10%, respectively, showing slightly better outcomes [10].

With regard to PBT for glioblastomas and other gliomas, there are currently four ongoing randomized controlled trials comparing PBT with photon therapy, however there are only a few single-center retrospective studies reporting outcomes in the published papers. Therefore, there are no reports of standard outcomes of PBT for glioblastomas. Mohan *et al.* reported a retrospective analysis of an ongoing randomized phase II trial comparing PBT with IMRT in glioblastomas, in which 60 Gy (RBE) in 30 fractions PBT with temozolomide was used, with a 2-year OS of approximately 50% in 28 patients [11]. In addition, a secondary analysis of PFS showed that the median PFS was 11.2 and 6.6 months for patients treated with PBT based on clinical radiological assessment and Response Assessment in Neuro-Oncology (RANO) criteria, respectively [12]. Adeberg *et al.* reported 63 cases of glioblastomas treated with 50–50.4 Gy in 25–28 fractions by X-rays with an additional 10 Gy (RBE) in five fractions of PBT, with a median OS of 19.1 months, 1- and 2-year OS of 72% and 40%, respectively, and median PFS of 8.8 months, 1- and 2-year PFS of 27% and 8%, respectively [13]. Mizumoto *et al.* reported that 46 patients were treated with 50.4 Gy in 28 fractions of X-rays plus 46.2 Gy (RBE) in 28 fractions of PBT given at least 6 hours apart on the same day for a total dose of 96.6 Gy in 56 fractions, with 1- and 2-year OS of 82.6% and 47.6%, respectively [14, 15]. Kong *et al.* reported results of particle therapy in 34 glioblastomas and 16 malignant gliomas: 24 patients were treated with 60 Gy (RBE) in 30 fractions of PBT, and another 26 patients were treated with PBT plus a carbon-ion radiotherapy boost to increase the dose above 60 Gy (RBE). The results showed that the 1- and 18-month OS for glioblastoma were 77.4% and 61% and the 1-year and 18-month PFS were 61.3% and 42.7%, respectively [16]. In all of these reports on PBT, there was concurrent administration of temozolomide, but it is not possible to simply compare treatment outcomes among these reports because the methods of radiation therapy delivery differ greatly, the evaluation indices other than the OS rate differ from report to report, and all are retrospective studies. In the present prospective observational study, the median survival time and specific survival rates were comparable to the standard treatment results with X-rays. However, due to the limited number of patients, the 95% confidence intervals are wide, making it difficult to statistically verify (argue for) the superiority or non-inferiority of PBT and photon radiation therapy, as in the case of common cancers, by using meta-analysis or other methods. However, since the treatment outcomes with PBT in these reports are also comparable to those reported by us, it is reasonable to presume that PBT is equivalent to photon therapy.

The physical characteristics of proton beams allow dose reductions in normal tissue while ensuring an adequate dose to the tumor. Several reports have found the superiority of the PBT dose distribution compared to X-rays for brain tumors, including gliomas [17–28]. Because malignant gliomas are rare and have a poor prognosis, it is difficult to fully evaluate late adverse events, but the acute adverse events of particle therapy in the above papers were all mild. Late adverse events ranged from 0–4.3% at Grade 3.

One of the 19 glioblastoma patients had Grade 3 central nervous system necrosis in this study. This patient was treated with hyperfractionated radiotherapy and a combination of X-rays and protons at 96.6 Gy (RBE) in 56 fractions. While none of the 16 patients who received 60 Gy (RBE) in 30 fractions of PBT developed Grade 3 or higher brain necrosis. This would support the suggestion that, although the number of patients is small and the follow-up period is short, PBT with standard dose fractionation appears able to reduce the risk of late adverse events, such as brain necrosis, cognitive dysfunction and others, and improve quality of life over that achieved with conventional radiotherapy using X-rays. The primary endpoint of a phase 2 trial conducted at The University of Texas MD Anderson Cancer Center (clinical trials.gov ID NCT01854554) is to evaluate time to cognitive failure between patients receiving intensity-modulated proton therapy and IMRT. Further, even the number of low-grade glioma patients was very small (two) in this study, the benefit of PBT may be expected to be greater because they are expected to have longer-term prognoses.

Of the patients included in this study, different doses from the JASTRO treatment policy of proton therapy were selected in two cases. One patient of clinically malignant glioma with a pathological diagnosis of oligodendroglioma received a total dose of 59.4 Gy (RBE) in 33 fractions after discussion by the tumor board. The other patient of diffuse astrocytoma was treated with 50.4 Gy (RBE) in 28 fractions because of its proximity to the optic chiasm. The consensus of the European Particle Therapy Network (EPTN) mentions that “For particles, most investigators also confirmed that the incidence of radiation induced optic neuropathy was low for a Dmax <54 Gy (RBE)”. They also set the dose constraint for the optic chiasm as D_0.03 cc_ ≤ 55 Gy with EQD2 [29]. On the other hand, there are several papers of the RBE varying and being higher than 1.1 depending on the relative position to the Bragg peak [30–34]. This patient is a case of diffuse astrocytoma. Although the JASTRO treatment policy of proton therapy indicates a treatment indication of 54 Gy (RBE) in 30 fractions, which would also satisfy the EPTN recommendation, considering the patient has a low-grade glioma and the possibility of radiation induced optic neuropathy due to the uncertainty of the RBE, clinical decision of 50.4 Gy (RBE) in 28 fractions seems reasonable.

There are several limitations to this study. First, the number of patients was small, and the study was observational and not adjusted for confounding factors. Second, the follow-up period was short and the evaluation of late adverse events was not sufficient, and the target volume of PBT was not specified in the JASTRO treatment policy of PBT. In addition, although this is a prospective study, there are many missing data in the molecular diagnostic information, especially the MGMT promoter methylation status. In the literature compared in this study, the diagnosis was based on WHO 2007 (IARC 4th edition) or earlier, and here further development of a diagnostic system is required to enable diagnosis in accordance with the current WHO classification.

In conclusion, we report on the current status of PBT for gliomas in Japan. For glioblastomas, which account for the majority of malignant gliomas, the outcomes of PBT are estimated to be equivalent to historical data of conventional radiotherapy using X-rays. The results of a prospective comparative evaluation of PBT and photon therapy are awaited.

## Data Availability

The data that support the findings of this study are available from Proton-Net. Restrictions apply to the availability of these data, which were used under license for this study, so supporting data is not available.

## CLINICAL TRIAL REGISTRATION NUMBER

UMIN000022917.

## References

[1] Brain Tumor Registry of Japan (2005–2008). Neurol Med Chir (Tokyo) 2017;57(Suppl 1):9-102. 28420810 10.2176/nmc.sup.2017-0001 PMC6760096

[2] Walker MD, Alexander E Jr, Hunt WE et al. Evaluation of mithramycin in the treatment of anaplastic gliomas. J Neurosurg 1976;44:655–67. 178838 10.3171/jns.1976.44.6.0655

[3] Andersen AP . Postoperative irradiation of glioblastomas. Results in a randomized series. Acta Radiol Oncol Radiat Phys Biol 1978;17:475–84. 216238 10.3109/02841867809128178

[4] Walker MD, Strike TA, Sheline GE. An analysis of dose-effect relationship in the radiotherapy of malignant gliomas. Int J Radiat Oncol Biol Phys 1979;5:1725–31. 231022 10.1016/0360-3016(79)90553-4

[5] Walker MD, Alexander E Jr, Hunt WE et al. Evaluation of BCNU and/or radiotherapy in the treatment of anaplastic gliomas. A cooperative clinical trial. J Neurosurg 1978;49:333–43. 355604 10.3171/jns.1978.49.3.0333

[6] Stupp R, Mason WP, van den Bent MJ et al. Radiotherapy plus concomitant and adjuvant temozolomide for glioblastoma. N Engl J Med 2005;352:987–96. 15758009 10.1056/NEJMoa043330

[7] Stupp R, Hegi ME, Mason WP et al. Effects of radiotherapy with concomitant and adjuvant temozolomide versus radiotherapy alone on survival in glioblastoma in a randomised phase III study: 5-year analysis of the EORTC-NCIC trial. Lancet Oncol 2009;10:459–66. 19269895 10.1016/S1470-2045(09)70025-7

[8] Stupp R, Taillibert S, Kanner AA et al. Maintenance therapy with tumor-treating fields plus Temozolomide vs Temozolomide alone for glioblastoma: a randomized clinical trial. JAMA 2015;314:2535–43. 26670971 10.1001/jama.2015.16669

[9] Rusthoven CG, Carlson JA, Waxweiler TV et al. The impact of adjuvant radiation therapy for high-grade gliomas by histology in the United States population. Int J Radiat Oncol Biol Phys 2014;90:894–902. 25585784 10.1016/j.ijrobp.2014.07.046

[10] Wakabayashi T, Natsume A, Mizusawa J et al. JCOG0911 INTEGRA study: a randomized screening phase II trial of interferonβ plus temozolomide in comparison with temozolomide alone for newly diagnosed glioblastoma. J Neuro-Oncol 2018;138:627–36. 10.1007/s11060-018-2831-7 PMC5999164 29557060

[11] Mohan R, Liu AY, Brown PD et al. Proton therapy reduces the likelihood of high-grade radiation-induced lymphopenia in glioblastoma patients: phase II randomized study of protons vs photons. Neuro-Oncology 2021;23:284–94. 32750703 10.1093/neuonc/noaa182 PMC7906048

[12] Al Feghali KA, Randall JW, Liu DD et al. Phase II trial of proton therapy versus photon IMRT for GBM: secondary analysis comparison of progression-free survival between RANO versus clinical assessment. Neurooncol Adv 2021;3:vdab073. 10.1093/noajnl/vdab073 PMC8320688 34337411

[13] Adeberg S, Bernhardt D, Harrabi SB et al. Sequential proton boost after standard chemoradiation for high-grade glioma. Radiother Oncol 2017;125:266–72. 29050959 10.1016/j.radonc.2017.09.040

[14] Mizumoto M, Yamamoto T, Takano S et al. Long-term survival after treatment of glioblastoma multiforme with hyperfractionated concomitant boost proton beam therapy. Pract Radiat Oncol 2015;5:e9–16. 25413424 10.1016/j.prro.2014.03.012

[15] Mizumoto M, Yamamoto T, Ishikawa E et al. Proton beam therapy with concurrent chemotherapy for glioblastoma multiforme: comparison of nimustine hydrochloride and temozolomide. J Neuro-Oncol 2016;130:165–70. 10.1007/s11060-016-2228-4 27535747

[16] Kong L, Wu J, Gao J et al. Particle radiation therapy in the management of malignant glioma: early experience at the shanghai proton and heavy ion Center. Cancer 2020;126:2802–10. 32167589 10.1002/cncr.32828 PMC7317504

[17] Amsbaugh MJ, Zhu XR, Palmer M et al. Spot scanning proton therapy for craniopharyngioma. Pract Radiat Oncol 2012;2:314–8. 24674170 10.1016/j.prro.2012.01.001

[18] Archambeau JO, Slater JD, Slater JM et al. Role for proton beam irradiation in treatment of pediatric CNS malignancies. Int J Radiat Oncol Biol Phys 1992;22:287–94. 1310964 10.1016/0360-3016(92)90045-j

[19] Boehling NS, Grosshans DR, Bluett JB et al. Dosimetric comparison of three-dimensional conformal proton radiotherapy, intensity-modulated proton therapy, and intensity-modulated radiotherapy for treatment of pediatric craniopharyngiomas. Int J Radiat Oncol Biol Phys 2012;82:643–52. 21277111 10.1016/j.ijrobp.2010.11.027

[20] Brower JV, Indelicato DJ, Aldana PR et al. A treatment planning comparison of highly conformal radiation therapy for pediatric low-grade brainstem gliomas. Acta Oncol 2013;52:594–9. 23421953 10.3109/0284186X.2013.767474 PMC3665211

[21] Eaton BR, Yock T. The use of proton therapy in the treatment of benign or low-grade pediatric brain tumors. Cancer J 2014;20:403–8. 25415686 10.1097/PPO.0000000000000079

[22] MacDonald SM, Safai S, Trofimov A et al. Proton radiotherapy for childhood ependymoma: initial clinical outcomes and dose comparisons. Int J Radiat Oncol Biol Phys 2008;71:979–86. 18325681 10.1016/j.ijrobp.2007.11.065

[23] Merchant TE, Hua CH, Shukla H et al. Proton versus photon radiotherapy for common pediatric brain tumors: comparison of models of dose characteristics and their relationship to cognitive function. Pediatr Blood Cancer 2008;51:110–7. 18306274 10.1002/pbc.21530

[24] Shih HA, Sherman JC, Nachtigall LB et al. Proton therapy for low-grade gliomas: results from a prospective trial. Cancer 2015;121:1712–9. 25585890 10.1002/cncr.29237 PMC12895348

[25] Tatsuzaki H, Urie MM, Linggood R. Comparative treatment planning: proton vs. x-ray beams against glioblastoma multiforme. Int J Radiat Oncol Biol Phys 1992;22:265–73. 1310962 10.1016/0360-3016(92)90043-h

[26] Yoon M, Shin DH, Kim J et al. Craniospinal irradiation techniques: a dosimetric comparison of proton beams with standard and advanced photon radiotherapy. Int J Radiat Oncol Biol Phys 2011;81:637–46. 20932690 10.1016/j.ijrobp.2010.06.039

[27] Halasz LM, Bussière MR, Dennis ER et al. Proton stereotactic radiosurgery for the treatment of benign meningiomas. Int J Radiat Oncol Biol Phys 2011;81:1428–35. 20934263 10.1016/j.ijrobp.2010.07.1991

[28] Kosaki K, Ecker S, Habermehl D et al. Comparison of intensity modulated radiotherapy (IMRT) with intensity modulated particle therapy (IMPT) using fixed beams or an ion gantry for the treatment of patients with skull base meningiomas. Radiat Oncol 2012;7:44. 10.1186/1748-717X-7-44 PMC3338385 22439607

[29] Lambrecht M, Eekers DBP, Alapetite C et al. Radiation dose constraints for organs at risk in neuro-oncology; the European particle therapy network consensus. Radiother Oncol 2018;128:26–36. 29779919 10.1016/j.radonc.2018.05.001

[30] Lühr A, von Neubeck C, Krause M et al. Relative biological effectiveness in proton beam therapy - current knowledge and future challenges. Clin Transl Radiat Oncol 2018;9:35–41. 29594249 10.1016/j.ctro.2018.01.006 PMC5862688

[31] Giantsoudi D, Adams J, MacDonald SM et al. Proton treatment techniques for posterior fossa Tumors: consequences for linear energy transfer and dose-volume parameters for the brainstem and organs at risk. Int J Radiat Oncol Biol Phys 2017;97:401–10. 27986346 10.1016/j.ijrobp.2016.09.042

[32] Underwood T, Paganetti H. Variable proton relative biological effectiveness: how do we move forward? Int J Radiat Oncol Biol Phys 2016;95:56–8. 27084627 10.1016/j.ijrobp.2015.10.006

[33] Peeler CR, Mirkovic D, Titt U et al. Clinical evidence of variable proton biological effectiveness in pediatric patients treated for ependymoma. Radiother Oncol 2016;121:395–401. 27863964 10.1016/j.radonc.2016.11.001 PMC5450501

[34] Maeda K, Yasui H, Matsuura T et al. Evaluation of the relative biological effectiveness of spot-scanning proton irradiation in vitro. J Radiat Res 2016;57:307–11. 26838131 10.1093/jrr/rrv101 PMC4915538

